# Context-specific effects of musical expertise on audiovisual integration

**DOI:** 10.3389/fpsyg.2014.01123

**Published:** 2014-10-01

**Authors:** Laura Bishop, Werner Goebl

**Affiliations:** ^1^Austrian Research Institute for Artificial Intelligence (OFAI)Vienna, Austria; ^2^Institute of Music Acoustics, University of Music and Performing Arts ViennaVienna, Austria

**Keywords:** musical expertise, multisensory integration, action prediction, ensemble performance, interpersonal coordination

## Abstract

Ensemble musicians exchange auditory and visual signals that can facilitate interpersonal synchronization. Musical expertise improves how precisely auditory and visual signals are perceptually integrated and increases sensitivity to asynchrony between them. Whether expertise improves sensitivity to audiovisual asynchrony in all instrumental contexts or only in those using sound-producing gestures that are within an observer's own motor repertoire is unclear. This study tested the hypothesis that musicians are more sensitive to audiovisual asynchrony in performances featuring their own instrument than in performances featuring other instruments. Short clips were extracted from audio-video recordings of clarinet, piano, and violin performances and presented to highly-skilled clarinetists, pianists, and violinists. Clips either maintained the audiovisual synchrony present in the original recording or were modified so that the video led or lagged behind the audio. Participants indicated whether the audio and video channels in each clip were synchronized. The range of asynchronies most often endorsed as synchronized was assessed as a measure of participants' sensitivities to audiovisual asynchrony. A positive relationship was observed between musical training and sensitivity, with data pooled across stimuli. While participants across expertise groups detected asynchronies most readily in piano stimuli and least readily in violin stimuli, pianists showed significantly better performance for piano stimuli than for either clarinet or violin. These findings suggest that, to an extent, the effects of expertise on audiovisual integration can be instrument-specific; however, the nature of the sound-producing gestures that are observed has a substantial effect on how readily asynchrony is detected as well.

## 1. Introduction

Prediction is central to success on joint action tasks. Whether two people are playing a piano duet or carrying a piano from one location to another, successful coordination hinges on each person being able to predict what the other's actions will be and what outcomes the combination of their own and their partner's actions will produce. Investigation of the mechanisms underlying prediction is particularly relevant in the context of music ensemble performance because of the precision and complexity of interpersonal coordination that is required. The present study investigated how the ability to predict others' actions is affected by expertise in music performance and how generalizable the effects of expertise are across instrumental contexts. With experience, musicians learn which types of actions will produce which types of sounds. Musicians' abilities to predict their own actions improve with increasing expertise (Keller and Koch, 2008; Bishop et al., 2013), and some research suggests that strengthened action-perception associations facilitate the prediction of others' actions as well (Aglioti et al., 2008; Petrini et al., 2009b; Wöllner and Cañal Bruland, 2010).

During ensemble performance, musicians receive auditory and visual signals that can aid prediction of their co-performers' actions (Williamon and Davidson, 2002; Ginsborg and King, 2009; Davidson, 2012). The amount of auditory and visual information available during ensemble performance is immense, and to make sense of it musicians must be able to bind together signals produced by the same event. Perceptual integration of auditory and visual signals is more likely to occur when those signals share temporal or semantic properties (Arrighi et al., 2006; Petrini et al., 2010a; Cook et al., 2011). People tolerate some asynchrony between auditory and visual signals. While the ear transmits incoming auditory information to the brain more rapidly than the eye transmits incoming visual information, differences in the transit times of light and sound mean that observers typically receive the visual signal from an event prior to receiving the auditory signal (Burr and Alais, 2006). People tend to rate audiovisual stimuli as most highly synchronized when the audio signal lags slightly behind the visual signal (Arrighi et al., 2006; Petrini et al., 2009a, 2010a). Audiovisual integration is a malleable process, subject to influence from both short-term and long-term experiences. In the short-term, several minutes' exposure to a particular degree of audiovisual asynchrony can prompt recalibration of sensory processing mechanisms, altering observers' perceptions of synchrony (Fujisaki et al., 2004; Vroomen et al., 2004). In the longer term, observers' sensitivity to audiovisual asynchrony relates to their familiarity with the presented stimuli (Vatakis and Spence, 2006; Saygin et al., 2008) and how readily they can predict the events in a perceived stimulus sequence (Petrini et al., 2009b; Cook et al., 2011; Lee and Noppeney, 2011). For instance, when presented with point-light displays of drumming actions containing arm but no drumstick markers, skilled percussionists are able to detect audiovisual asynchronies, while novices are not (Petrini et al., 2009b). Percussionists' familiarity with drumming actions likely enables them to predict drumstick-drumhead contact points and the corresponding sound onsets based on observed arm trajectories. They are then able to identify sounds that do not occur at the expected time relative to predicted drumstick-drumhead contact points. Novices, in contrast, do not predict drumstick trajectories or sound onsets as precisely and, as a result, are less sensitive to asynchronies between them. Thus, observers' prediction abilities both influence and are influenced by the effectiveness of audiovisual integration. In the present study, musicians' sensitivities to audiovisual asynchrony in videos of instrumental performance were assessed as a way of investigating their prediction abilities.

Some research suggests that the effects of perceptual-motor expertise on audiovisual integration may be context-specific. Lee and Noppeney (2011) found pianists to be more sensitive than non-musicians to audiovisual asynchrony in videos of piano playing. Using fMRI, they examined brain regions involved in asynchrony detection and, comparing pianists and non-musicians, observed enhanced activity among pianists in a network comprising superior temporal sulcus, premotor, and cerebellar areas during the presentation of piano stimuli. In contrast, pianists and non-musicians showed similar patterns of brain activation when presented with videos of a person speaking, and did not differ in their abilities to detect audiovisual asynchrony in speech stimuli. These results suggest that piano performance experience relates specifically to improved temporal processing during the perception of piano playing and does not generalize to speech perception. Whether the effects of musical expertise on audiovisual integration generalize across instrumental contexts remains unclear, however. In previous studies investigating the effects of expertise on audiovisual integration, musician groups have been homogeneous with regards to the type of instrumentalists included, comprising only pianists (Lee and Noppeney, 2011) or percussionists (Petrini et al., 2009b, 2010a, 2011). Different instruments make markedly different motor demands on performers, and experience in performing the actions involved in playing one instrument may not improve prediction of the actions involved in playing other instruments. In the present study, musicians' sensitivities to audiovisual asynchrony in clarinet, piano, and violin performance were assessed. Pairings between participants' instrument of expertise (clarinet, piano, or violin/viola) and the instruments featured in stimulus videos were manipulated so that the generalizability of musicians' sensitivities to audiovisual asynchrony across instrumental contexts could be assessed.

Evidence that musicians' prediction abilities may not generalize across instruments comes from their performance on synchronization tasks. Keller et al. (2007) found that skilled pianists synchronized more successfully with their own previously-recorded expressive performances than with performances recorded by other pianists. It was concluded that pianists predict co-performers' actions most successfully when those actions are part of the pianist's own motor repertoire and incoming information about others' actions can map directly onto the observer's own action system. Incoming visual information is thought to trigger the internal simulation of observed actions, with more direct mappings between observed actions and observers' action systems enabling more effective simulations and more accurate prediction of action outcomes (Calvo-Merino et al., 2006; Keller et al., 2007; Schubotz, 2007; Aglioti et al., 2008; Wöllner and Cañal Bruland, 2010; Keller, 2012; Novembre et al., 2012). With an improved ability to predict when co-performers' actions will occur, musicians are better able to time their own actions so that synchrony is achieved.

Extensive visual exposure to instrumental performance might be expected to improve musicians' prediction abilities just as physical performance experience does. Research has shown that motor skills can be learned through observation, in the absence of physical practice. Thus, the action-effect associations underlying prediction may strengthen without overt action execution (Mattar and Gribble, 2005; Cross et al., 2009; Brown and Palmer, 2012). Studies comparing visual and motor experts, however, suggest that visual and performance experience do not improve prediction abilities to the same extent (Aglioti et al., 2008). For example, musicians with both instrumental and conducing performance experience have been found to tap more precisely with single-beat conductor gestures than can either non-musicians or musicians with instrumental but no conducting experience (Luck and Nte, 2008). Though participants in both musician groups likely had extensive experience in observing and synchronizing with conductor gestures, musicians with practical conducting experience showed an advantage over those without. Performance expertise, therefore, may have a facilitatory effect on prediction above and beyond any effect of visual expertise.

Wöllner and Cañal Bruland (2010), similarly, found string musicians to synchronize with violinists' cueing-in gestures more accurately than either non-musicians or skilled non-string musicians. Participants were shown silent video clips depicting violinists' gestures and asked to synchronize a keypress with the implied note onset. In addition to responding more accurately, string musicians displayed lower timing variability than non-musicians or non-string musicians. Such a finding parallels the low variability that motor experts across domains show in executing well-practiced actions (Starkes and Allard, 1993; Davids et al., 2006). Thus, a motor system capable of carrying out particular actions with low variability may be capable of simulating others' similar actions with low variability as well. String musicians' superior performance relative to non-string musicians' suggests that the benefits of performance expertise may not transfer across instrumental contexts. If performance expertise had facilitated prediction regardless of the instrumental context, then string and non-string musicians would have shown a similar advantage over non-musicians. An alternative explanation is that performance expertise facilitates the prediction of violin gestures for reasons that are specific to the context of string instrument performance. For instance, it may be particularly difficult to extract timing information from bowing gestures—which are continuous and yield gradual note onsets—without prior experience in performing similar actions and hearing the effects. While string musicians have an advantage over non-string musicians when making predictions about violinists' gestures, perhaps non-string musicians have no such advantage over string musicians when making predictions about wind or keyboard performance. Clarinetists, pianists, and violinists were included in the present study so that the potential effects of performance expertise on audiovisual integration could be assessed in these different instrumental contexts.

The present study tested the hypothesis that music performance expertise improves observers' abilities to predict others' actions, increasing sensitivity to audiovisual asynchrony, but only in the context of instruments that the observer is experienced in playing. Clarinetists, pianists, and violinists/violists completed an audiovisual asynchrony detection task, which acted as an indirect measure of their prediction abilities. Pairings between observers' instrument of expertise and the instruments featured in stimulus videos (clarinet, piano, violin) were manipulated so that the generalizability of expertise effects across instrumental contexts could be assessed. First, highly-skilled clarinetists, pianists, and violinists performed three pieces for audio and video recording. They performed with piano accompaniment so that the use of visual gestures would be encouraged. Each clarinet-piano, piano-piano, and violin-piano duo recorded a “deadpan,” “normally-expressive,” and “overly-expressive” performance of each piece. A pilot experiment was conducted to verify that the video recordings did not differ in quality between instrument categories. During the main experiment, highly-skilled clarinetists, pianists, and violinists/violists were presented with short video clips extracted from the “normally-expressive” piano, violin, and clarinet performances, and asked to judge as quickly as possible whether the audio and video channels were synchronized. Clips either maintained the audiovisual synchrony present in the original recordings or were modified so that the video led or lagged behind the audio (total 9 levels of asynchrony).

It was hypothesized that musicians would be more sensitive to audiovisual asynchrony in performances featuring their own instrument than in performances featuring another instrument. In other words, clarinetists were expected to be most sensitive to audiovisual asynchrony in videos of clarinet performance, pianists were expected to be most sensitive to audiovisual asynchrony in videos of piano performance, and violinists were expected to be most sensitive to audiovisual asynchrony in videos of violin performance. All participants had extensive experience in ensemble performance and, therefore, extensive experience in observing the performance of other instruments. An effect of visual expertise on sensitivity to audiovisual asynchrony was not expected, however, in line with previous research suggesting that motor expertise facilitates action simulation to a greater extent than does visual expertise (Aglioti et al., 2008; Luck and Nte, 2008; Wöllner and Cañal Bruland, 2010).

## 2. Duo performance recordings

Seven highly-skilled musicians (two clarinetists, three pianists, two violinists) were recruited from the University of Music and Performing Arts Vienna to perform for audio and video recording. They had between 9 and 28 years of musical training (*M* = 17.1, *SD* = 6.5) and played regularly, performing between 5 and 50 times per year (*M* = 25.7, *SD* = 18.4). All but one clarinetist performed professionally; all had substantial prior experience in ensemble performance and reported playing as part of both large (e.g., full orchestra) and small (e.g., duo, trio, quartet) ensembles.

**Figure 1:**
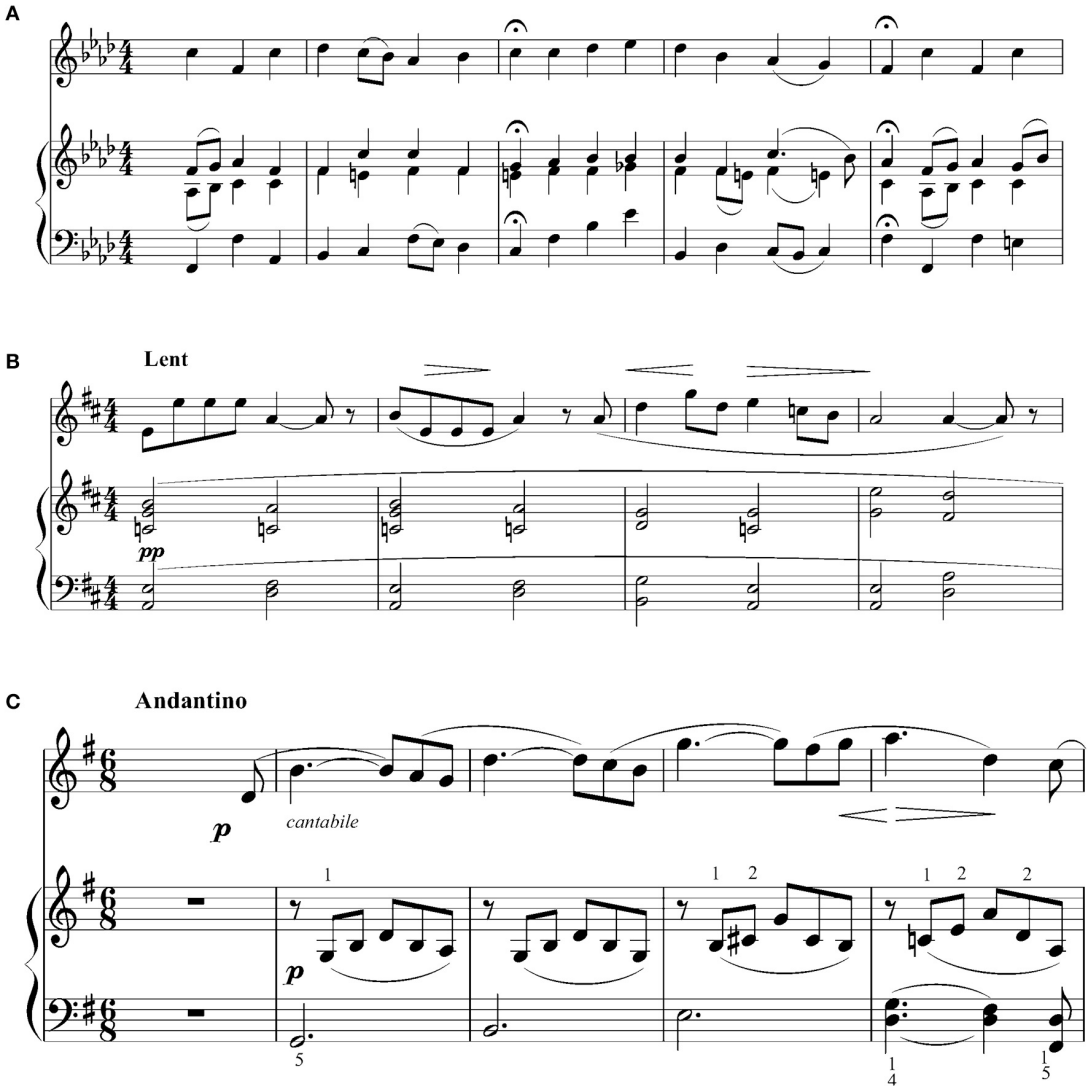
**Excerpts from experimental stimuli**. **(A)** “Aus tiefer Noth schrei ich zu dir,” **(B)** “Les Anges,” **(C)** *Winterabend*.

Recordings were made of each duo performing three pieces: *Kirchenmusik*, Op. 23, “Aus tiefer Noth schrei' ich zu dir,” by Felix Mendelssohn, *Trois Méelodies de 1886*, “Les Anges,” by Erik Satie, and *Winterabend*, by Ludvig Schytte (Figure 1). The piece by Mendelssohn is a chorale for four voices. For the current study, the alto, tenor, and bass lines were combined and performed by the piano accompanist, and the soprano line was performed by the clarinet, piano, or violin soloist. Some slight alterations were made to the accompanist's part to simplify the voice-leading. The piece by Satie is a song for one voice and piano; the accompanist played the piano part and the soloist played the vocal line. Slight alterations were made to the accompanist's part to eliminate large chords that most pianists would otherwise have rolled. Notes were also added to the soloist part in place of rests so that the soloist (who was the subject of the video recordings) could cue in the accompanist (who did not appear in the videos), rather than the accompanist cueing in the soloist. The piece by Schytte is a piano duet for four hands. The accompanist played the secondo part, and a single melody line was extracted from the primo part to be performed by the soloist. The structure of this piece was simplified by substituting the final melody section (bars 45–60 in the original score) for the introduction that makes up the first 24 bars of the original composition, thus giving the piece an ABA form. Some pitch alterations were also made to simplify the “B” section of the solo part.

These pieces were chosen because they represented a range of musical forms and differed from each other in character and tempo. Each piece was expected to present certain challenges to the duos that would encourage communication via non-verbal cues. The Mendelssohn piece, for instance, comprises seven phrases that each end with fermatas (i.e., long held notes in both soloist and accompanist parts). Non-verbal communication between performers was necessary to coordinate the release of these held notes and the onset of the following phrases. The Satie piece has a very slow tempo and would typically be played with a great deal of expressive timing and dynamics; performers were expected to use non-verbal cues to communicate their expressive intentions to each other and coordinate note onsets and offsets. In the Schytte piece, performers were expected to make particular use of non-verbal cues for communicating their expressive intentions and synchronizing chords that were meant to be played in unison (e.g., bars 34–37).

**Figure 2:**
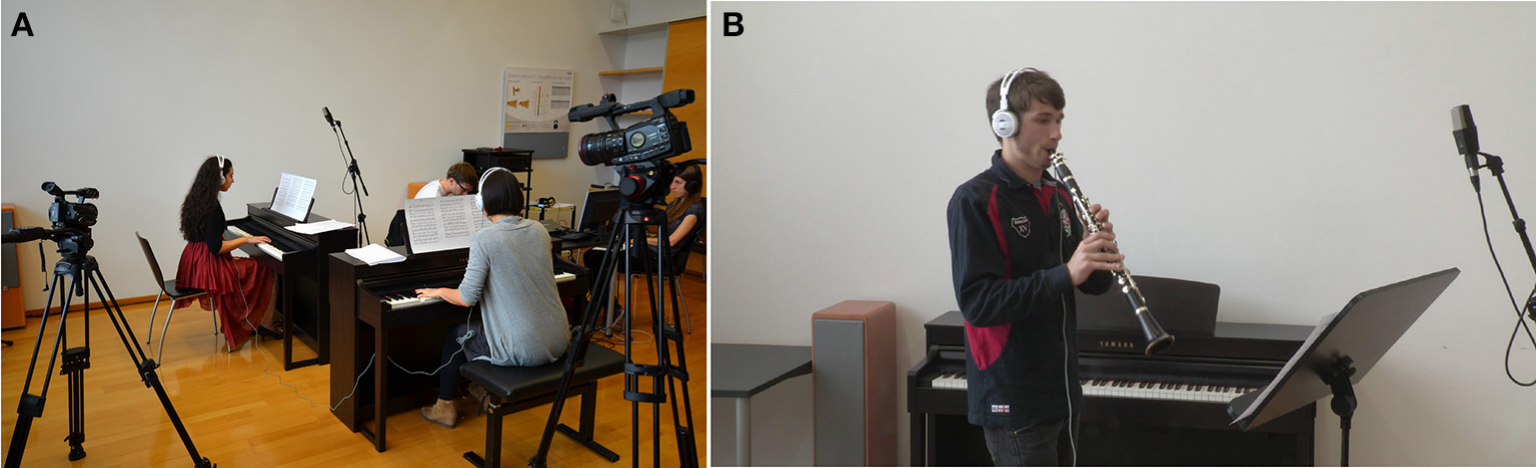
**Experimental stimuli**. **(A)** Camera set-up during recording of a pianist. The accompanist had a lateral view of the soloist (far left). **(B)** Sample frame from a lateral-view clarinet recording.

Recordings were made for each duo individually, using two video cameras. Recording sessions were overseen by a professional recording engineer. The soloist and accompanist were placed about two meters apart, with the accompanist facing the soloist directly and the soloist at a right angle to the accompanist's line of sight (Figure 2). One camera (Canon XF305) stood slightly behind the accompanist and captured a lateral view of the soloist. The other camera (Canon SDHC) stood to the rear right of the soloist, and captured the view from behind. The cameras recorded all performances simultaneously with a frame rate of 25 frames/s. During piano-piano duo performances, both soloist and accompanist played on Yamaha CLP-470 Clavinovas. During clarinet-piano and violin-piano duo performances, the soloist Clavinova was moved out of the way and the clarinetist or violinist stood in its place. Two pairs of AKG K520 (semi-open) headphones, connected to the accompanying Clavinova, allowed musicians to hear the accompanist's sound. Audio from the solo instrument played in the room and could be heard clearly by both performers despite their headphones. An AKG C414 B-ULS microphone and a Sound Devices 778T recorder were used to make audio recordings of soloists' performances. Audio from all soloist performances and MIDI data from soloist and accompanist Clavinovas were collected on a MacBook Pro. An Ambient Clockit Lanc Logger ALL 601 was connected to this computer and used to sync audio, video, and MIDI timecodes. The Lanc Logger sends a signal to other machines that are connected to the computer and synchronizes their real time to within 0.2 ppm, enabling precise audiovisual synchronization in recordings made using multiple devices.

Musicians provided written informed consent prior to beginning the recording session and agreed to the use of their videos as experimental stimuli. Recording procedures were in accordance with the Declaration of Helsinki (revised 1983). The musicians were given hard copies of the score for each piece at the start of the recording session. Scores displayed both solo and accompanist parts, and included any expressive notation (e.g., cues to dynamic or tempo changes; phrase markings) and fingering (for pianists) that had appeared on the original compositions. The accompanist was given a target tempo for each piece and used the Clavinova metronome function to play a couple bars of beats prior to each rehearsal of a piece. Musicians were given as much time as they needed to practice before performing for recording. It was stressed that the soloist was to lead performances, and the accompanist was to follow. Prior to the start of each recorded performance, the accompanist again set the tempo using the metronome. The metronome was then turned off, and the soloist cued the onset of the piece. Three versions of each piece were recorded: a “normally-expressive” version, which duos were instructed to play expressively, as practiced, a “deadpan” version, which they were instructed to play as mechanically as possible, without any expression, and an “overly-expressive” version, which they were instructed to play dramatically, with exaggerated expression. These expressive conditions were selected in order to obtain recordings of the same material, performed by the same musicians, that contained noticeable between-performance differences in gestures and sound. In the pilot experiment, audio and video channels from the deadpan and overly-expressive recordings were recombined. Participants viewed short video clips from the original and recombined recordings and judged the compatibility of audio and video channels.

## 3. Pilot experiment

The pilot experiment was conducted in order to check that clarinet, piano, and violin videos were equally informative to viewers, or in other words, that participants could make accurate judgements about the compatibility of audio and video channels in videos featuring the different stimulus instruments, with no one instrument yielding universally poor results. Short clips were extracted from the deadpan and overly-expressive recordings and presented either without manipulation or with audio and video channels recombined across performances. Clarinetists were expected to be most accurate at judging whether audio and video derived from the same performance for clarinet stimuli, while pianists were expected to be most accurate for piano stimuli and violinists were expected to be most accurate for violin stimuli.

### 3.1. Participants

Twelve musicians completed the pilot experiment (4 clarinetists, 4 pianists, 4 violinists/violists), none of whom had taken part in recording the duo performances. The musicians were between 20 and 30 years of age (*M* = 24.6, *SD* = 3.0). They reported between 7 and 20 years of training (*M* = 15.7, *SD* = 4.0) and were active musicians, practicing between 3 and 40 h per week (*M* = 19.9, *SD* = 13.4) and performing between 2 and 150 times per year (*M* = 36, *SD* = 41.6). All performed on their instrument professionally. Most currently played or had previous experience in playing other instruments, but they all considered the clarinet, piano, violin, or viola to be their primary instrument. All participants reported extensive experience in ensemble performance.

### 3.2. Materials and procedure

Short clips were extracted from each duo's deadpan and overly-expressive performances (Figure 2). Audio and video recordings were imported into Final Cut Pro and synced according to their timecodes. Audio-video clips were then selected from each performance. For half of the clips (“different” clips), the video excerpt from one performance was combined with the audio excerpt from the same duo's other performance—that is, video excerpts from deadpan performances were combined with audio from overly-expressive performances, and video excerpts from overly-expressive performances were combined with audio from deadpan performances. For the other half of the clips (“same” clips), audio and video from the same part of the same performance were presented without modification. Clips were saved as MOV files with a frame rate of 25 frames/s and audio sampling rate of 44.1 kHz, and were displayed to participants in a 960 by 540 pixel window on a computer screen.

For each duo, two locations per piece were selected and four clips were made at each location. At one location, deadpan audio was combined with deadpan video and presented at lateral (clip 1) and rear camera angles (clip 2), and deadpan audio was combined with overly-expressive video and presented at lateral (clip 3) and rear camera angles (clip 4). At the other location, overly-expressive audio was combined with overly-expressive video and presented at lateral (clip 5) and rear camera angles (clip 6), and overly-expressive audio was combined with deadpan video and presented at lateral (clip 7) and rear camera angles (clip 8). With eight clips per piece for each of the six duos, there were 144 clips made. During the experiment, 48 clips were shown twice so that internal reliability could be evaluated. Thus, the experiment comprised 192 trials. The clips extracted from duo performances ranged between 3.00 and 7.02 s in length (*M* = 5.13, *SD* = 1.52). Some, though not all, clips began and ended at phrase boundaries.

All participants provided written informed consent prior to beginning the experiment, and all experimental procedures were in accordance with the Declaration of Helsinki (revised 1983). They completed the task individually, seated at a PC and wearing AKG K520 headphones. A custom-made patch in Max/MSP presented the audio-video clips and collected participant response data, including response time. The 192 clips were presented in four blocks of 48 trials, with participants encouraged to pause between blocks. Each participant saw the clips in one of five prescribed, pseudo-random orders, which were constrained so that the “same” and “different” versions of a clip were never presented in immediate succession. Participants were asked to view each clip and indicate as quickly as possible whether the audio and video were from the same performance or different performances by pressing one of two marked keys on the computer keyboard. If they did not respond within 3 s of the end of the clip, a notice appeared on the screen reminding them to respond quickly. The question participants were to respond to remained visible on the screen throughout the experiment: Do the sound and image come from the same performance or different performances? (“Stammen Bild und Ton von der selben Aufnahme oder von verschiedenen?”). Boxes on the screen marked same (“der selben”) and different (“verschiedenen”) lit up whenever a participant made the corresponding response. The number of trials remaining in a block was always visible on the screen as well. After completing the task, participants were asked to respond to some questions about their musical background. The experiment took approximately 40 min to complete.

### 3.3. Results and discussion

**Figure 3:**
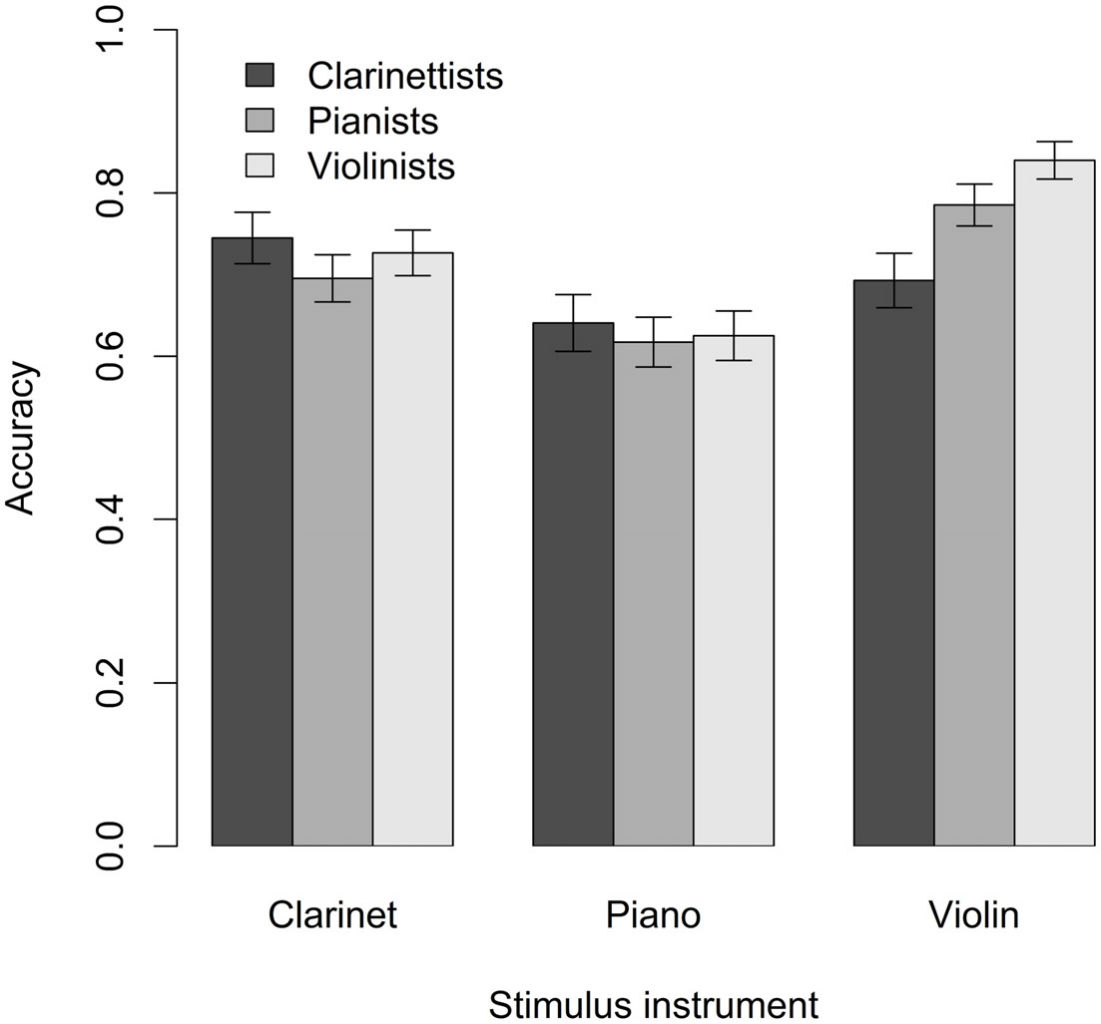
**Pilot task response accuracy**. Error bars indicate standard error.

Figure 3 shows clarinetists', pianists', and violinists' accuracy at detecting audio-video mismatches in clarinet, piano, and violin stimuli. It was hypothesized that musicians would detect audio-video mismatches more successfully for performances featuring the instrument they were experienced in playing than for performances featuring other instruments. To investigate the potential relationship between performance expertise and stimulus instrument, a 3 × 3 repeated-measures ANOVA was conducted using expertise group (clarinet, piano, violin/viola) as the between-subject independent variable, stimulus instrument (clarinet, piano, violin) as the within-subject independent variable, and mean response accuracy as the dependent variable. A significant main effect of stimulus instrument was observed, *F*_(2, 21)_ = 4.6, *p* = 0.02. There was no significant main effect of expertise group, *F*_(2, 21)_ = 1.2, *p* = 0.31, and no significant interaction between motor expertise and stimulus instrument, *F*_(4, 21)_ = 1.4, *p* = 0.27. Post-tests showed that with data pooled across expertise groups, participants performed significantly better for violin stimuli than for clarinet and piano stimuli combined, *t*_(10)_ = 3.3, *p* = 0.01 (using a Bonferroni-adjusted alpha of 0.02). They also performed significantly worse for piano stimuli than for clarinet and violin stimuli combined, *t*_(10)_ = 5.4, *p* < 0.001. Their performance for clarinet stimuli did not differ from their performance of violin and piano stimuli combined, *t*_(10)_ = 0.8, *p* = 0.46.

A subset of the stimulus set was presented to participants twice during the experiment, so that consistency in their responses could be assessed. Phi-coefficients, which measure the association between two binary variables, were calculated to evaluate the similarity between participants' first and second responses to these items. A small but significant positive correlation was observed with data pooled across participants and stimuli, *ϕ* = 0.18, *p* < 0.001. Within stimulus instrument categories, a significant correlation was achieved only for violin stimuli, *ϕ* = 0.17, *p* < 0.001. Participants did not respond with as high consistency to either clarinet, *rϕ* = 0.08, *p* = 0.30, or piano stimuli, *ϕ* = 0.05, *p* = 0.45.

These results suggest that participants detected audiovisual mismatches most readily in violin stimuli and least readily in piano stimuli. Different types and magnitudes of movement are needed to play the clarinet, piano, and violin. The larger-scale movements involved in violin bowing might provide more salient cues to expressive timing and dynamics than the smaller-scale hand movements involved in piano-playing. Many participants also reported monitoring the violin performances for vibrato, which was typically present in overly-expressive performances and absent in deadpan performances. Vibrato could not be used as a cue to audiovisual mismatches in piano stimuli. A potential explanation for participants' difficulties with piano stimuli might be that pianists do not realize their expressive intentions as well on a Clavinova as they do on an acoustic piano, but such a possibility requires further research.

The videos presented to participants in the pilot experiment featured lateral and rear camera angles. To determine whether camera angle had an effect on audiovisual mismatch detection accuracy, a 2 × 3 repeated measures ANOVA was run, using camera angle (lateral, rear) and stimulus instrument (clarinet, piano, violin) as within-subject independent variables and mean response accuracy as the dependent variable. A significant main effect of stimulus instrument was observed, *F*_(2, 56)_ = 6.0, *p* = 0.004, but there was no significant effect of camera angle, *F*_(1, 56)_ = 1.6, *p* = 0.21, and no interaction between camera angle and stimulus instrument, *F*_(2, 56)_ = 1.1, *p* = 0.35. To reduce the number of variables included in the main experiment, only lateral videos were used. The mean accuracy and standard deviation of each stimulus clip was calculated as well, in order to identify items that participants across expertise groups judged with below-chance accuracy. There were twelve stimulus clips (8% of the stimulus pool) that participants judged with low accuracy (10–35% of responses to these items were correct). All were “different” clips, and low accuracy was achieved because participants erroneously judged them to be the same. In case participants' low accuracy for these items related to issues with the musical material, main experiment stimulus clips were not selected from these potentially-problematic locations in the music.

The pilot experiment investigated whether clarinet, piano, and violin stimuli were similarly informative to clarinetist, pianist, and violinist viewers. Participants responded least accurately to piano stimuli regardless of their instrumental expertise, suggesting that the piano stimuli were less informative than the clarinet and violin stimuli. The main experiment assessed audiovisual asynchrony detection and was, therefore, quite a different task from the pilot, which also required participants to detect discrepancies between observed actions and sound quality. Since pianists might extract timing information from videos of piano performance more readily than they extract information about dynamics or tone, pianists were retained, and the main experiment again tested clarinetist, pianist, and violinist/violist groups.

## 4. Main experiment

The main experiment investigated the potential instrument-specific effects of expertise on sensitivity to audiovisual asynchrony among highly-skilled clarinetists, pianists, and violinists. Participants judged the audiovisual synchrony in clips from clarinet, piano, and violin performances and were expected to be most sensitive to asynchrony in clips featuring the instrument they could play.

### 4.1. Participants

Thirty-eight musicians (24 female) completed the experiment. Ten of these participants had previously completed the pilot experiment, but none had performed for the recordings. Twelve clarinetists, fourteen pianists, and twelve violinists/violists were tested, but two pianists were subsequently excluded because they did not complete the experiment as instructed. Thus, data for 36 participants were included in the analyses. All participants had extensive experience in ensemble performance. The clarinetists (age *M* = 23.1, *SD* = 3.6) reported an average of 11.8 years of training (*SD* = 5.2), 19.8 h of practice per week (*SD* = 8.5), and 55 concert performances per year (*SD* = 62); the pianists included in the analyses (age *M* = 24.3, *SD* = 4.1) reported an average of 16.6 years of training (*SD* = 2.6), 24.4 h of practice per week (*SD* = 14.7), and 21 concert performances per year (*SD* = 14.7); the violinists (age *M* = 23.7, *SD* = 5.0) reported an average of 14.3 years of training (*SD* = 4.5), 29.8 h of practice per week (*SD* = 7.7), and 31 concerts per year (*SD* = 18).

### 4.2. Materials and procedure

One clip per piece was selected from each duo's normally-expressive performance recordings. As in the pilot experiment, audio and video recordings were imported into Final Cut Pro and their timecodes were synced. Clips were selected from the same locations in the music as in the pilot experiment, but were longer in length, ranging between 8.02 and 10.22 s (*M* = 9.75, *SD* = 0.8). In contrast to the pilot experiment, for all clips used in the main experiment, both audio and video came from the recordings of normally-expressive performances. Eight asynchronous versions of each clip were constructed by shifting the video track either forwards or back with respect to the audio by 1, 3, 5, and 7 frames (corresponding to 40, 120, 200, and 280 ms). A version of each clip that preserved the audio-visual synchrony present in the original recordings (i.e., to which no audio or video delay was introduced) was also made. All versions of a clip thus contained the same audio segment, differing only in the video segment that was shown. As in the pilot, clips were saved as MOV files with a frame rate of 25 frames/s and an audio sampling rate of 44.1 kHz, and presented in a 960 by 540 pixel window on a computer screen. With three clips selected for each of the six duos, and each clip presented at nine levels of asynchrony, 162 items were displayed during the experiment. One-third of these items were presented twice so that internal reliability could be assessed. The experiment, therefore, contained 216 trials.

At the start of the session, the task was explained and participants provided written informed consent. The experiment was conduced in accordance with the Declaration of Helsinki (revised 1983). Participants completed the task individually, using the same PC and AKG K520 headphones as were used in the pilot experiment. A custom-made patch in Max/MSP presented the audiovisual stimuli in a random order and collected participant response data, including response time. Participants completed the task in 4 blocks of 54 trials, with a break between each block. They were asked to view each item and indicate as quickly as possible whether or not the audio and video were synchronized by pressing one of two marked buttons on the computer keyboard. As in the pilot experiment, a notice appeared on the computer screen reminding them to respond faster if they failed to respond within 3 s of the end of a clip. The question to which participants were to respond (“Are audio and video synchronized”; “Sind Bild und Ton synchronisiert?”) and the number of trials remaining in the block remained on the screen throughout the experiment. Boxes labeled synchronized (“synchronisiert”) and not synchronized (“nicht synchronisiert”) lit up whenever the participant pressed the corresponding response key. At the end of the experiment, participants completed a musical background questionnaire. The experiment took approximately 1 h to complete.

Due to problems with the original testing machine, three computers were used for data collection: 21 participants completed the experiment on a Dell Inspiron running Windows 7 (7 in each expertise group), 15 used a Macbook Pro running OS × 10.8.5 (5 in each expertise group), and 2 used an HP Ultrabook running Windows 7 (both pianists). A test was run to assess how these machines differed in the temporal precision of their audiovisual display. A 60-s clip was extracted from one of the performance recordings. The original audio was removed in Final Cut Pro and replaced with a click track (one click every 500 ms). The original video was modified to include a square that alternated between black and white at 500 ms intervals, in synchrony with the auditory clicks. The video file was exported in MOV format, just as experimental stimuli had been. This modified video was then played on each computer using the Max/MSP patch designed for the main experiment. An EG and G Vactec VT935G photoresistor was attached to the computer screen, positioned over the square that had been added to the video, and registered the discrete changes in lightness. As the video played, audio (the click track) and output from the photoresistor were captured using a Waveterminal U2A audio interface and recorded in separate channels in Audacity. Data from three trials were collected for each computer. The onset time of each click and corresponding visual change was identified manually in Audacity, and audiovisual asynchronies were calculated. Averaged across trials, the mean asynchrony was +33.7 ms (*SD* = 26.4) for the Dell (the positive asynchrony indicating that audio lagged behind video), −96.9 ms (*SD* = 28.5) for the MacBook (with video lagging behind audio), and +46.1 ms (*SD* = 22.5) for the HP. The difference in audiovisual asynchrony between computers was significant, *F*_(2, 1048)_ = 3265.3, *p* < 0.001, but the variability was not, *F*_(1, 7)_ = 3.5, *p* = 0.10. Each participant's measured point of subjective synchrony was adjusted according to the testing computer used, in order to correct for the effects of computer-specific asynchrony (see below).

## 5. Results

Trials with very long response times were excluded from the analyses. Data were pooled across participants and stimulus categories, and 11 trials with response times further than 3 *SD* from the overall mean were identified as outliers. These excluded times can be assumed to indicate trials on which participants interrupted the experiment, as they ranged between 19.7 and 153.7 s in length, which is substantially more than a participant would have spent deliberating over an answer. Some trials (288 total, or 3.5%, divided among 7 participants) were also lost due to technical problems.

**Figure 4:**
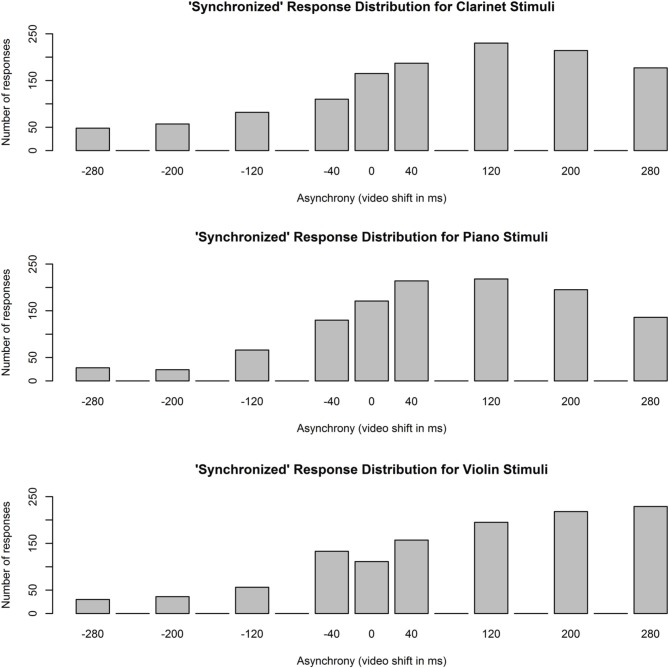
**Distributions of “synchronized” responses for clarinet, piano, and violin stimuli**. Data have been pooled across expertise groups and have not been changed to reflect the adjustment that was subsequently applied to PSS values. At positive asynchronies, audio lagged behind video; at negative asynchronies, video lagged behind audio. Only asynchrony values with bars and labels were included in the experiment; empty bins correspond to delays that were not tested.

An interaction between participant expertise group and stimulus instrument was predicted, with participants expected to demonstrate the greatest sensitivity to audiovisual asynchrony in performances on their own instrument. Two measures were calculated for each participant's responses to clarinet, piano, and violin stimuli: (1) the Point of Subjective Synchrony (PSS), or the category of asynchrony that a participant most often rated as synchronized, and (2) the Temporal Integration Window (TIW), or the range of asynchronies the participant most often rated as synchronized. These measures are commonly reported in the literature on audiovisual integration (Arrighi et al., 2006; Petrini et al., 2009a; Lee and Noppeney, 2011). Typically, Gaussian curves are fit to participant distributions of “synchronized” responses, with curve peaks used as a measure of PSS and standard deviation used as a measure of TIW. In contrast to previous studies, however, the current study used a between-subject rather than within-subject design in order to test for potential differences between types of instrumentalists. Participants received only nine stimulus presentations at each delay category, which is too few data points for curves to be fit to the response distributions of individual participants. Instead, the mode of each participant response distribution (i.e., the asynchrony category receiving the highest number of “synchronized” responses) was used as a measure of PSS, and the interquartile range (IQR; i.e., the range of asynchronies that received 50% of a participant's “synchronized” responses) was used as a measure of TIW. Mode is a more representative measure of central tendency than weighted mean or median would have been, and IQR is a more representative measure of spread than standard deviation. Response distributions for each stimulus instrument, with data pooled across expertise groups, are shown in Figure 4. Shapiro-Wilk tests indicated that these distributions were significantly different from normal for clarinet, *W* = 0.93, *p* < 0.001, piano, *W* = 0.95, *p* < 0.001, and violin stimuli, *W* = 0.91, *p* < 0.001. Thus, the range of audiovisual asynchronies musicians accepted as synchronized for clarinet, piano, and violin stimuli was not centered within the range of tested delays.

To account for effects of computer-specific asynchrony on PSS magnitude, PSS values were linearly shifted for each participant by the mean asynchrony observed on the computer they used to complete the experiment: 34 ms were added to clarinet, piano, and violin PSS values for participants who used the Dell, 97 ms were subtracted from PSS values for participants who used the MacBook, and 46 ms were added to PSS values for participants who used the HP. Though they affected PSS magnitude, these computer-specific asynchronies would not have altered the effects of either stimulus instrument or expertise on PSS. A greater number of participants completed the experiment on the Dell than on the MacBook, but in both cases participants were evenly distributed across expertise groups (and only two participants used the HP).

### 5.1. Point of subjective synchrony

**Figure 5:**
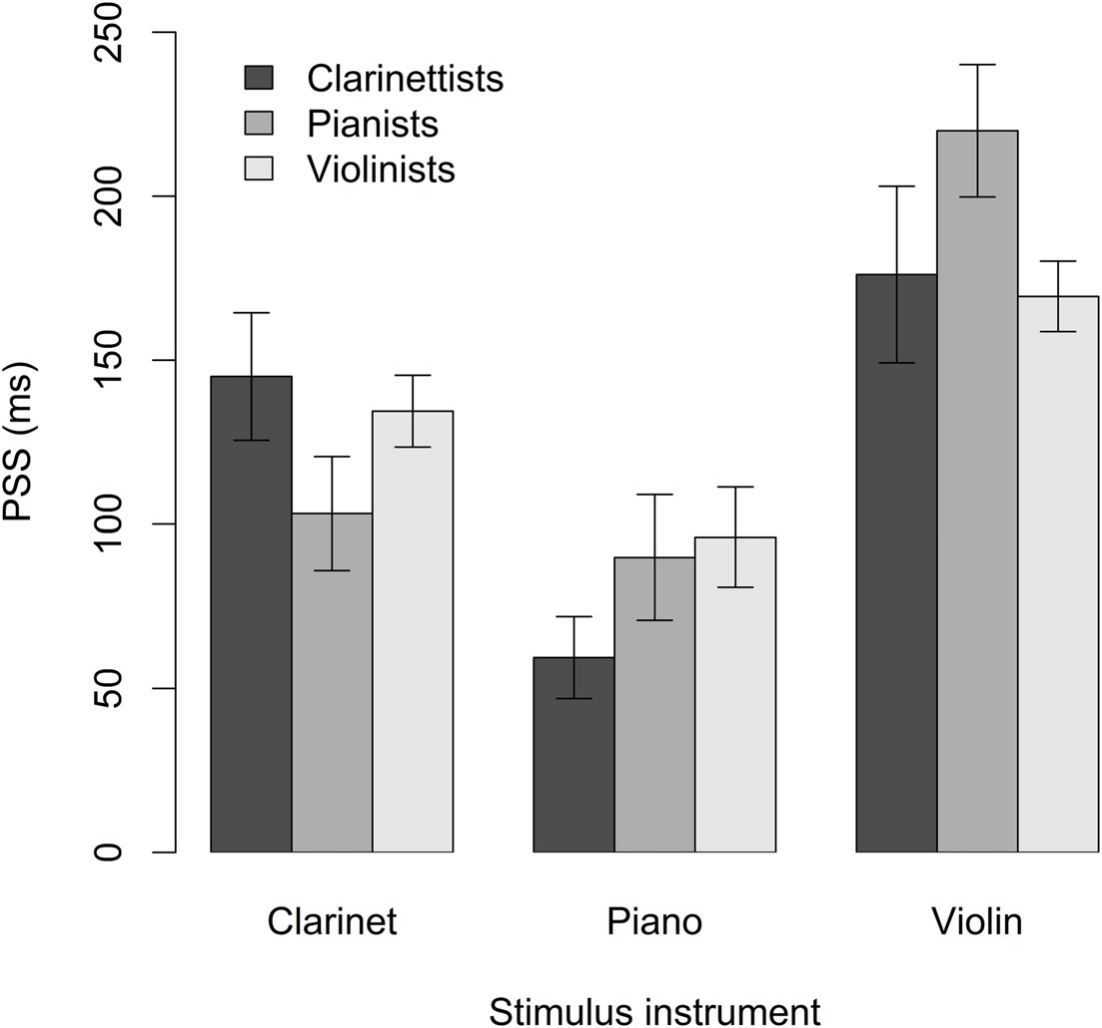
**Mean PSS across stimulus instruments and expertise groups**. Error bars indicate standard error. At positive PSS values, audio lagged behind video.

The average PSS values for clarinetists, pianists, and violinists responding to clarinet, piano, and violin videos are shown in Figure 5. For all expertise/stimulus instrument combinations, PSS values were positive, indicating that stimuli in which the audio lagged behind the video were perceived as synchronized. Linear mixed-effects modeling (LME) was conducted using the “nlme” package in R (R Core Team, 2013) to assess the effects of participant expertise and stimulus instrument on PSS. Mixed-effects models test a combination of fixed effects, or effects attributable to manipulated variables, and random effects, or effects attributable to specified sources of random error (e.g., subjects or experimental items) (Barr et al., 2012). Main effects and interactions between predictors can be tested as with ANOVA. In the case of a repeated-measures design, the lack of independence between observations can be accounted for by nesting subjects within predictors.

Participants' PSS values for clarinet, piano, and violin stimuli were modeled using the interaction between expertise group and stimulus instrument as the fixed component and subjects nested within stimulus instrument as the random component. The model showed a significant main effect of stimulus instrument, *F*_(2, 66)_ = 38.3, *p* < 0.001, and a significant interaction between stimulus instrument and expertise group, *F*_(4, 66)_ = 3.4, *p* = 0.01. There was no main effect of expertise, *F*_(2, 33)_ = 0.19, *p* = 0.83. The PSS occurred closer to 0 ms asynchrony for piano than for clarinet and violin stimuli combined, *t*_(66)_ = 7.2, *p* < 0.001, and further from 0 ms asynchrony for violin than for clarinet and piano combined, *t*_(66)_ = 7.9, *p* < 0.001 (Table 1).

**Table 1:** **Mean PSS and TIW (*SD*)**.

**Stimulus**	**PSS (ms)**	**TIW (ms)**
Clarinet	+128 (58)	124 (35)
Piano	+82 (56)	106 (31)
Violin	+188 (72)	127 (40)

*PSS and TIW values have been averaged across all participants. Positive values indicate that audio lagged behind video*.

Planned contrasts were run to compare PSS values within expertise groups, between stimulus instrument categories, to further investigate the interaction between expertise and stimulus instrument. Clarinetists' mean PSS was no different for clarinet and violin stimuli, but they showed a significantly lower mean PSS for piano than for clarinet stimuli, *z* = 4.0, *p* < 0.001. Pianists' mean PSS was no different for piano and clarinet stimuli, but they showed a significantly lower mean PSS for piano than for violin stimuli, *z* = 6.1, *p* < 0.001. Violinists' mean PSS was no different for violin and clarinet stimuli, but they also showed a significantly lower mean PSS for piano than for violin stimuli, *z* = 3.5, *p* = 0.001. Planned contrasts comparing PSS values across expertise groups, within stimulus instrument categories, did not yield any significant results. Thus, for all expertise groups, PSS values were closest to 0 ms audio delay for piano stimuli and tended to be furthest from 0 ms audio delay for violin stimuli. PSS, in this experiment, seems to have been determined primarily by characteristics of the stimuli instruments and influenced little by participants' expertise.

### 5.2. Temporal integration window

**Figure 6:**
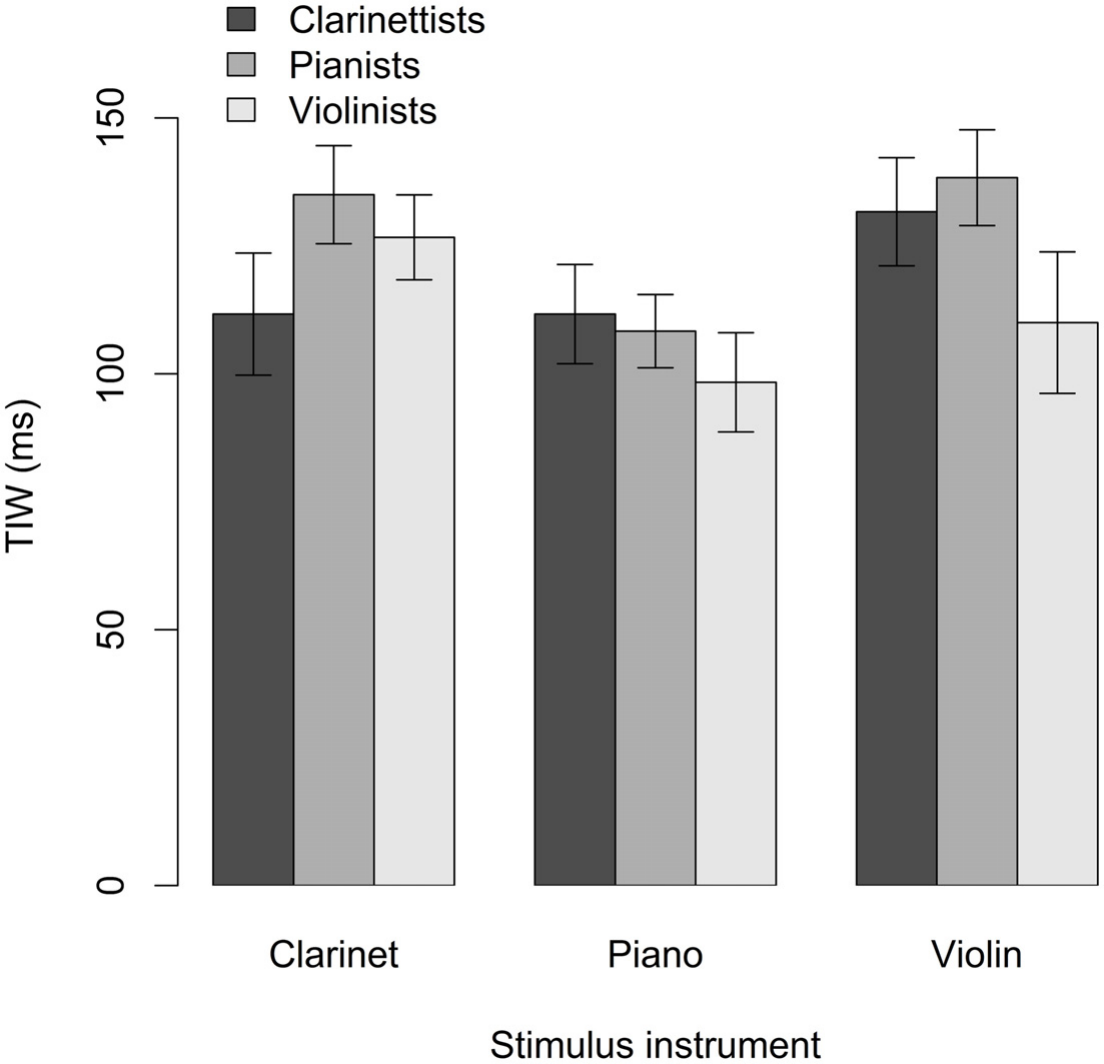
**Mean TIW across stimulus instruments and expertise groups**. Error bars indicate standard error.

Mean TIW values for clarinetists', pianists', and violinists' responses to clarinet, piano, and violin videos are shown in Figure 6. Participants' TIW values for clarinet, piano, and violin stimuli were modeled using the interaction between expertise group and stimulus instrument as the fixed variable component and subjects nested within stimulus instrument as the random variable component, as in the analysis for PSS. The model yielded a significant main effect of stimulus instrument, *F*_(2, 66)_ = 6.5, *p* = 0.003. Neither the main effect of expertise, *F*_(2, 33)_ = 0.94, *p* = 0.40, nor the interaction between expertise and stimulus instrument was significant, *F*_(4, 66)_ = 1.9, *p* = 0.12. Mean TIW was found to be smaller for piano stimuli than for a combination of clarinet and violin stimuli, *t*_(66)_ = 3.6, *p* < 0.001, and larger for violin stimuli than for a combination of clarinet and piano stimuli, *t*_(66)_ = 2.1, *p* = 0.04 (Table 1).

Planned contrasts comparing TIW within expertise groups, between stimulus instrument categories, showed that pianists had a narrower TIW for piano stimuli than for clarinet stimuli, which approached significance at a Bonferroni-adjusted alpha of 0.02, *z* = 2.5, *p* = 0.03, and a significantly narrower TIW for piano stimuli than for violin stimuli, *z* = 2.8, *p* = 0.01. Clarinetists' TIW values did not differ significantly between either clarinet and piano, *z* = 0.0, *p* > 0.99, or clarinet and violin stimuli, *z* = 1.9, *p* = 0.12. Likewise, violinists' TIW values did not differ significantly between either violin and clarinet, *z* = 1.5, *p* = 0.21, or violin and piano stimuli, *z* = 1.1, *p* = 0.45. Planned contrasts were also run to compare TIW across expertise groups, within stimulus instrument categories, but none of these contrasts was significant.

On the other hand, when data were pooled across expertise groups and stimulus instrument categories, a significant negative correlation was observed between TIW and years of formal musical training, *r* = −0.28, *p* = 0.004, indicating that TIW decreased with increasing musical experience. This finding suggests that musical training relates to improved sensitivity to audiovisual asynchrony in musical stimuli regardless of the instrumental context. The results also provide some evidence that instrument-specific expertise may have affected participants' performance: pianists were more sensitive to asynchrony in piano than either clarinet or violin stimuli. Clarinetists, likewise, tended to be more sensitive than pianists or violinists to asynchrony in clarinet stimuli, and violinists tended to be more sensitive than clarinetists or pianists to asynchrony in violin stimuli, though these tendencies did not reach significance. Musicians across expertise groups also showed narrower TIWs for piano than for either clarinet or violin stimuli, suggesting that sensitivity to audiovisual asynchrony may be affected by stimulus instrument characteristics.

### 5.3. Response consistency and perceptual recalibration effects

Fifty-four of the 162 unique stimulus videos were presented to participants twice, so that the consistency of their responses could be assessed. Phi coefficients were calculated to evaluate the consistency in participants' responses across the first and second viewings of these items. A correlation was calculated for each asynchrony category with data pooled across participants. Correlation values ranged between *ϕ* = 0.10 and *ϕ* = 0.38 and were significant (all *p* < 0.01) in eight of the nine asynchrony categories. The only correlation that did not reach significance was in the +120 ms audio lag category, suggesting that, overall, participants were least reliable in their responses at this degree of asynchrony. Otherwise, participants were consistent in their judgements throughout the experiment.

Short-term exposure to asynchronous audiovisual stimuli has been shown to influence observers' perceptions of simultaneity through a recalibration of sensory processing mechanisms (see Fujisaki et al., 2004; Vroomen et al., 2004). While effects of short-term exposure were not expected in the current experiment, since stimuli from different delay categories were only 10 s in length and presented to each participant in a random order, a check was made to ensure that this was the case. We tested whether participant responses depended on the delay category of the previous trial by running Chi-squared tests on the response distributions for each stimulus instrument category (pooled across all participants). The results were not significant for clarinet, χ^2^(8, *N* = 1989) = 10.9, *p* = 0.21, piano, χ^2^(8, *N* = 1873) = 5.1, *p* = 0.74, or violin stimuli, χ^2^(8, *N* = 1811) = 9.5, *p* = 0.30, indicating that the asynchrony present in one trial did not affect participants' perceptions of synchrony during the next trial. We also tested whether participant responses depended on the delay category of the current trial by running Chi-squared tests on the responses distributions shown in Figure 4. Significant results were found for clarinet, χ^2^(8, *N* = 1989) = 290.2, *p* < 0.001, piano, χ^2^(8, *N* = 1873) = 376.1, *p* < 0.001, and violin stimuli, χ^2^(8, *N* = 1811) = 362.0, *p* < 0.001, indicating that participants' perceptions of synchrony were affected by the delay present in the current trial stimulus.

## 6. General discussion

Perceptual-motor expertise relates to improved prediction of observed actions (e.g., Luck and Nte, 2008; Petrini et al., 2009a; Wöllner and Cañal Bruland, 2010; Lee and Noppeney, 2011). The present study investigated to what extent the effects of musical expertise on audiovisual integration are instrument-specific. Clarinetists, pianists, and violinists made synchrony judgements for video clips extracted from clarinet, piano, and violin performances, and the potential effects of performance expertise on participants' points of subjective synchrony and temporal integration windows were assessed. TIW was found to improve with increasing musical training, indicating an effect of musical expertise on sensitivity to audiovisual asynchrony that aligns with previous research. Some support was found for the hypothesis that the effects of expertise on sensitivity to audiovisual asynchrony are instrument-specific, as pianists performed better on piano stimuli than on either clarinet or violin stimuli. There was also a tendency for clarinetists to outperform others on clarinet stimuli and violinists to outperform others on violin stimuli, but these differences did not reach significance. A main effect of stimulus instrument was significant for both measures, with PSS closest to 0 ms for piano stimuli and furthest from 0 ms for violin stimuli. TIW was likewise narrowest for piano stimuli and widest for violin stimuli. Sensitivity to audiovisual asynchrony, therefore, was highest for piano stimuli and lowest for violin stimuli, independent of expertise group. This finding suggests characteristics specific to particular instruments, such as the type of sound-producing movements used and corresponding differences in attack time (see Gordon, 1987), influence precision in audiovisual integration. Thus, this study provides evidence that sensitivity to audiovisual asynchrony is influenced by expertise and instrument characteristics, and it provides some evidence that the influence of expertise can be instrument-specific.

While prior research has investigated differences between expert musicians and non-musicians, the main question addressed in the present study was whether the effects of musical expertise would be instrument-specific. Some expert abilities, including facets of musical imagery ability, have previously been found to generalize to unfamiliar motor contexts (Keller and Koch, 2008; Bishop et al., 2014). Since predicting the outcomes of one's own and others' actions draws on imagery ability (Keller and Appel, 2010; Keller et al., 2010; Bishop et al., 2013), tasks assessing prediction could be expected to yield generalized rather than instrument-specific expertise effects as well. Pianists in the present study were more sensitive to audiovisual asynchrony in piano stimuli than in either clarinet or violin stimuli, however, suggesting that to some extent, the influence of musical expertise on audiovisual integration may be instrument-specific. On the other hand, the same effect was not found for clarinetists or violinists, even though both groups tended to perform better than others on videos showing their own instrument. Instrument-specific expertise effects might have been attenuated in the present study by an effect of visual expertise. All participants had substantial experience in observing performances on other instruments as a result of their ensemble experience. While in previous research, motor expertise has been shown to facilitate prediction to a greater extent than does visual expertise, these studies have used synchronization tasks rather than audiovisual asynchrony detection tasks (Luck and Nte, 2008; Wöllner and Cañal Bruland, 2010). In the context of a synchronization task, which requires participants to pair their own overt actions with the actions they are observing, the effects of visual expertise may be small, while in the context of an audiovisual asynchrony judgment task, which does not require the execution of overt, precisely-timed actions, the effects of visual expertise may be greater.

A main effect of stimulus instrument was observed for both PSS and TIW, with participants across groups showing a narrower mean TIW and PSS closer to 0 ms asynchrony for piano stimuli, and a wider mean TIW and PSS further from 0 ms asynchrony for violin stimuli. In prior research investigating the effects of musical expertise on sensitivity to audiovisual asynchrony, drumming stimuli were used because the salience of the movements and rapid onsets of the sounds were expected to make detecting asynchronies relatively easy (Love et al., 2012). PSS values obtained in the present study were substantially larger than those obtained in these previous studies, likely due in part to the increased complexity of the auditory and visual stimuli used, and in part to characteristics of the stimulus instruments. The overall mean PSS for violin stimuli was particularly large, falling at the largest degree of audio delay that was tested. Thus, even for skilled musicians, the threshold for detecting audiovisual asynchronies in violin performance seems to be high. Note onsets and offsets are often gradual in string instrument performance, and the movements involved in bowing are more continuous than the percussive finger movements used to depress keys on the piano or clarinet. These properties may render subtle audiovisual asynchronies in violin performance less perceptible. Sensitivity to asynchrony was also lower for clarinet than piano stimuli, perhaps because the small finger movements used to depress keys are more critically linked to sound production on the piano. In clarinet performance, sound onsets and offsets are ultimately a result of breathing and tonguing, which may offer less salient cues than finger movements (Hofmann and Goebl, 2014). Effects of expertise may also have contributed to the differences observed between stimulus instruments, across expertise groups. All students at the University of Music and Performing Arts Vienna receive piano instruction; seven of the clarinetists and nine of the clarinetists reporting at least 2 years of formal piano training (violinists range 2–10; clarinetists range 2–15). This prior piano training might have facilitated the performance of some clarinetists and violinists on piano stimuli. In contrast, only one clarinetist and one pianist reported any violin training, and no violinists or pianists played the clarinet.

In contrast to the main experiment, where participants were most sensitive overall to audiovisual asynchrony in piano stimuli, in the pilot experiment, participants were least accurate in detecting audiovisual mismatches in piano stimuli. In a study investigating musicians' evaluations of piano performance recordings, in which a single soundtrack accompanied either the corresponding video performance or the video for another pianist's performance of the same piece, Behne and Wöllner (2011), likewise, found that musicians failed to notice discrepancies between audio and video channels. An acoustic rather than digital instrument was used to construct stimuli, suggesting that participants' inabilities to detect audiovisual mismatches in the present study might not be attributable to our use of a Clavinova and the disruptions to the relationship between movement and sound quality that may result. Our pilot task required participants to attend to expressive parameters such as dynamics, articulation, and vibrato, while the main experiment required attention only to timing. The difference between pilot and main experiment results suggests that musicians may extract temporal information from videos of piano performance more readily than they extract information about other expressive parameters. Prior research suggests that people can make accurate judgements about the emotional quality of music despite asynchrony between audio and visual signals (Petrini et al., 2010b). The auditory modality tends to dominate over the visual modality when people are asked to make judgements about perceived musical expression, though the visual signal can confirm or accentuate the audio signal when the signals are congruous (Vines et al., 2006; Petrini et al., 2010b). Separate brain networks may be used for evaluating temporal synchrony and sensory congruency (e.g., differences between movement velocity and sound intensity) in audiovisual stimuli (Petrini et al., 2011). The two tasks used in the present study may, therefore, have engaged two different neural systems. Further research would be needed to investigate why one system would have more difficulty with piano than clarinet or violin stimuli, while the other system had less.

To achieve a coordinated performance, ensemble musicians must be able to detect when sounds from their co-performers' instruments fail to occur at the expected time. When predicted and perceived timing differ, musicians may need to adjust the timing of their own actions to re-establish coordination. Deviations between predicted and perceived timing might also sometimes indicate that a musician has misinterpreted a co-performer's auditory or visual timing cues, and should attend more carefully to avoid further erroneous predictions. Investigation of the relationship between musical expertise and sensitivity to audiovisual asynchrony is particularly significant in the context of networked music performance. Current collaborative software systems enable musicians to perform together in real-time while in remote locations; however, network connection problems can arise, causing delays in the transfer of audio and/or video from one musician to another (Bartlette et al., 2006). If these network latencies are substantial, they can interfere with musicians' abilities to coordinate their actions. When there is an audience who may be watching and listening from yet another location, network latencies can reduce their perception of a coherent performance. The present study shows that the nature of sound-producing movements—whether they are continuous, as in violin bowing, or percussive, as in piano-playing—affect how readily audiovisual asynchronies are detected by highly-skilled musicians. Effects of expertise on sensitivity to audiovisual asynchrony were observed, and the results suggest that to some extent, the effects of expertise can be instrument-specific. Participants with less performance expertise were likely less precise than participants with more performance experience in predicting the relative timing of auditory and visual signals. Less-experienced musicians may also have had less explicit knowledge about where to direct their visual attention when judging the audiovisual synchrony of observed performances. Such findings suggest that more experienced musicians may be more impaired by audiovisual asynchrony in co-performers' signals during ensemble performance than are less experienced musicians. Future research should investigate whether the effects of expertise on audiovisual integration differ in magnitude across categories of sound-producing movement, as well as exploring the impact musicians' sensitivities to asynchrony have on their ensemble performance.

## Funding

This research was supported by the Austrian Science Fund (FWF) grant P24546.

### Conflict of interest statement

The authors declare that the research was conducted in the absence of any commercial or financial relationships that could be construed as a potential conflict of interest.

## References

[B1] Aglioti S. M. Cesari P. Romani M. Urgesi C. (2008). Action anticipation and motor resonance in elite basketball players. Nat. Neurosci. 11, 1109–1116 10.1038/nn.2182 19160510

[B2] Arrighi R. Alais D. Burr D. (2006). Perceptual synchrony of audiovisual streams for natural and artificial motion sequences. J. Vis. 6, 260–268 10.1167/6.3.6 16643094

[B3] Barr D. J. Levy R. Scheepers C. Tily H. J. (2012). Random effects structure for confirmatory hypothesis testing: keep it maximal. J. Mem. Lang. 68, 255–278 10.1016/j.jml.2012.11.001 24403724 PMC3881361

[B4] Bartlette C. Headlam D. Bocko M. Velikic G. (2006). Effect of network latency on interactive musical performance. Music Percept. 24, 49–62 10.1525/mp.2006.24.1.49

[B5] Behne K. E. Wöllner C. (2011). Seeing or hearing the pianists? a synopsis of an early audiovisual perception experiment and a replication. Music. Sci. 15, 324–342 10.1177/1029864911410955

[B6] Bishop L. Bailes F. Dean R. T. (2013). Musical imagery and the planning of dynamics and articulation during performance. Music Percept. 31, 97–116 10.1525/mp.2013.31.2.97

[B7] Bishop L. Bailes F. Dean R. T. (2014). Performing musical dynamics: how crucial are musical imagery and auditory feedback for expert and novice musicians? Music Percept. 32, 51–66 10.1525/mp.2014.32.1.51

[B8] Brown R. M. Palmer C. (2012). Auditory-motor learning influences auditory memory for music. Mem. Cogn. 40, 567–578 10.3758/s13421-011-0177-x 22271265

[B9] Burr D. Alais D. (2006). Combining visual and auditory information. Prog. Brain Res. 155B, 243–258 10.1016/S0079-6123(06)55014-9 17027392

[B10] Calvo-Merino B. Grezes J. Glaser D. E. Passingham R. E. Harrad P. (2006). Seeing or doing? influence of visual and motor familiarity in action observation. Curr. Biol. 16, 1905–1910 10.1016/j.cub.2006.07.065 17027486

[B11] Cook L. A. Van Valkenburg D. L. Badcock D. R. (2011). Predictability affects the perception of audiovisual synchrony in complex sequences. Attent. Percept. Psychophys. 73, 2286–2297 10.3758/s13414-011-0185-8 21800221

[B12] Cross E. S. Kraemer D. J. M. Hamilton A. Kelley W. M. Grafton S. T. (2009). Sensitivity of the action observation network to physical and observational learning. Cereb. Cortex 19, 315–326 10.1093/cercor/bhn083 18515297 PMC2638791

[B13] Davids K. Bennett S. Newell K. (2006). Movement System Variability. Champaign, IL: Human Kinetics

[B14] Davidson J. W. (2012). Bodily movement and facial actions in expressive musical performance by solo and duo instrumentalists: two distinctive case studies. Psychol. Music 40, 595–633 10.1177/0305735612449896

[B15] Fujisaki W. Shimojo S. Kashino M. Nishida S. (2004). Recalibration of audiovisual simultaneity. Nat. Neurosci. 7, 773–778 10.1038/nn1268 15195098

[B16] Ginsborg J. King E. (2009). Gestures and glances: the effects of familiarity and expertise on singers' and pianists' bodily movements in ensemble rehearsals, in 7th Triennial Conference of European Society for the Cognitive Sciences of Music, eds Louhivuori J. Eerola T. Saarikallio S. Himberg T. Eerola P. (Jyväskylä).

[B17] Gordon J. W. (1987). The perceptual attack time of musical tones. J. Acoust. Soc. Am. 82, 88–105 10.1121/1.395441 3624645

[B18] Hofmann A. Goebl W. (2014). Production and perception of legato, portato, and staccato articulation in saxophone playing. Front. Psychol. 5:690 10.3389/fpsyg.2014.00690 25076918 PMC4097958

[B19] Keller P. E. (2012). Mental imagery in music performance: underlying mechanisms and potential benefits. Ann. N.Y. Acad. Sci. 1252, 206–213 10.1111/j.1749-6632.2011.06439.x 22524361

[B20] Keller P. E. Appel M. (2010). Individual differences, auditory imagery, and the coordination of body movements and sounds in musical ensembles. Music Percept. 28, 27–46 10.1525/mp.2010.28.1.27

[B20a] Keller P. E. Dalla Bella S. Koch I. (2010). Auditory imagery shapes movement timing and kinematics: evidence from a musical task. J. Exp. Psychol. Hum. Percept. Perform. 36, 508–513 10.1037/a0017604 20364934

[B22] Keller P. E. Knoblich G. Repp B. (2007). Pianists duet better when they play with themselves: on the possible role of action simulation in synchronization. Conscious. Cogn. 16, 102–111 10.1016/j.concog.2005.12.004 16466932

[B23] Keller P. E. Koch I. (2008). Action planning in sequential skills: relations to music performance. Q. J. Exp. Psychol. 61, 275–291 10.1080/17470210601160864 17853237

[B24] Lee H. Noppeney U. (2011). Long-term music training tunes how the brain temporally binds signals from multiple senses. Proc. Natl. Acad. Sci. U.S.A. 108, 1441–1450 10.1073/pnas.1115267108 22114191 PMC3251069

[B25] Love S. Pollick F. Petrini K. (2012). Effects of Experience, Training and Expertise on Multisensory Perception: Investigating the Link Between Brain and Behavior. Berlin; Heidelberg: Springer, Dresden

[B26] Luck G. Nte S. (2008). An investigation of conductors' temporal gestures and conductor-musician synchronization, and a first experiment. Psychol. Music 36, 81–99 10.1177/0305735607080832

[B27] Mattar A. A. G. Gribble P. L. (2005). Motor learning by observation. Neuron 46, 153–160 10.1016/j.neuron.2005.02.009 15820701

[B28] Novembre G. Ticini L. Schütz-Bosbach S. Keller P. (2012). Distinguishing self and other in joint action: evidence from a musical paradigm. Cereb. Cortex 22, 2894–2903 10.1093/cercor/bhr364 22235034

[B29] Petrini K. Dahl S. Rocchesso D. Waadeland C. H. Avanzini F. Puce A. (2009a). Multisensory integration of drumming actions: musical expertise affects perceived audiovisual asynchrony. Exp. Brain Res. 198, 339–352 10.1007/s00221-009-1817-2 19404620

[B30] Petrini K. Holt S. P. Pollick F. (2010a). Expertise with multisensory events eliminates the effect of biological motion rotation on audiovisual synchrony perception. J. Vis. 10, 1–14 10.1167/10.5.2 20616132

[B30b] Petrini K. McAleer P. Pollick F. (2010b). Audiovisual integration of emotional signals from music improvisation does not depend on temporal correspondence. Brain Res. 1323, 139–148 10.1016/j.brainres.2010.02.012 20153297

[B32] Petrini K. Pollick F. Dahl S. McAleer P. McKay C. Rocchesso D. (2011). Action expertise reduces brain activity for audiovisual matching actions: an fMRI study with expert drummers. Neuroimage 56, 1480–1492 10.1016/j.neuroimage.2011.03.009 21397699

[B29a] Petrini K. Russell M. Pollick F. (2009b). When knowing can replace seeing in audiovisual integration of actions. Cognition 110, 432–439 10.1016/j.cognition.2008.11.015 19121519

[B34] R Core Team. (2013). R: A Language and Environment for Statistical Computing. Vienna: R Foundation for Statistical Computing

[B35] Saygin A. P. Driver J. de Sa V. R. (2008). In the footsteps of biological motion and multisensory perception: judgments of audiovisual temporal relations are enhanced for upright walkers. Psychol. Sci. 19, 469–475 10.1111/j.1467-9280.2008.02111.x 18466408

[B36] Schubotz R. I. (2007). Prediction of external events with our motor system: towards a new framework. Trends Cogn. Sci. 11, 211–218 10.1016/j.tics.2007.02.006 17383218

[B37] Starkes J. L. Allard F. (1993). Cognitive Issues in Motor Expertise. Amsterdam: Elsevier Science

[B38] Vatakis A. Spence C. (2006). Audiovisual synchrony perception for music, speech, and object actions. Brain Res. 1111, 134–142 10.1016/j.brainres.2006.05.078 16876772

[B39] Vines B. Krumhansl C. Wanderley M. Levitin D. (2006). Cross-modal interactions in the perception of musical performance. Cognition 101, 80–113 10.1016/j.cognition.2005.09.003 16289067

[B40] Vroomen J. Keetels M. de Gelder B. Bertelson P. (2004). Recalibration of temporal order perception by exposure to audio-visual asynchrony. Cogn. Brain Res. 22, 32–35 10.1016/j.cogbrainres.2004.07.003 15561498

[B41] Williamon A. Davidson J. W. (2002). Exploring co-performer communication. Music. Sci. 6, 53–72 10.1177/102986490200600103

[B42] Wöllner C. Cañal Bruland R. (2010). Keeping an eye on the violinist: motor experts show superior timing consistency in a visual perception task. Psychol. Res. 74, 579–585 10.1007/s00426-010-0280-9 20300943 PMC2938444

